# Supplementary material for: Microorganisms Involved in Hydrogen Sink in the Gastrointestinal Tract of Chickens
Source: Int J Mol Sci. 2023 Apr 3;24(7):6674. doi: 10.3390/ijms24076674 (PMC10095559; doi:10.3390/ijms24076674)
Supplement: Supplementary file 1 [file ijms-24-06674-s001.zip › table S1.pdf]

Pair of variables	All chicken groups together The Spearman rho's rank correlation results; *p* < 0.05000		
	R_s Spearman	t(N-2)	*p*
Methanogenic archaea (log10) & Methanogenic archaea (log10)			
Methanogenic archaea (log10) & Acetogens (log10)	0.055560	0.69724	0.486686
Methanogenic archaea (log10) & Sulfate-reducing bacteria (log10)	0.551535	8.28472	0.000000
Methanogenic archaea (log10) & Hydrogenase utilizers (log10)	0.464421	6.57077	0.000000
Methanogenic archaea (log10) & *L. salivarius* (log10)	0.049604	0.62230	0.534646
Methanogenic archaea (log10) & *C. jejuni* (log10)	0.472977	6.72632	0.000000
Acetogens (log10) & Methanogenic archaea (log10)	0.055560	0.69724	0.486686
Acetogens (log10) & Acetogens (log10)			
Acetogens (log10) & Sulfate-reducing bacteria (log10)	-0.337500	-4.49245	0.000014
Acetogens (log10) & Hydrogenase utilizers (log10)	0.163816	2.08071	0.039084
Acetogens (log10) & *L. salivarius* (log10)	-0.143439	-1.81606	0.071268
Acetogens (log10) & *C. jejuni* (log10)	0.014771	0.18511	0.853386
Sulfate-reducing bacteria (log10) & Methanogenic archaea (log10)	0.551535	8.28472	0.000000
Sulfate-reducing bacteria (log10) & Acetogens (log10)	-0.337500	-4.49245	0.000014
Sulfate-reducing bacteria (log10) & Sulfate-reducing bacteria (log10)			
Sulfate-reducing bacteria (log10) & Hydrogenase utilizers (log10)	0.241187	3.11400	0.002194
Sulfate-reducing bacteria (log10) & *L. salivarius* (log10)	0.263881	3.42791	0.000777
Sulfate-reducing bacteria (log10) & *C. jejuni* (log10)	0.431407	5.99177	0.000000
Hydrogenase utilizers (log10) & Methanogenic archaea (log10)	0.464421	6.57077	0.000000
Hydrogenase utilizers (log10) & Acetogens (log10)	0.163816	2.08071	0.039084
Hydrogenase utilizers (log10) & Sulfate-reducing bacteria (log10)	0.241187	3.11400	0.002194
Hydrogenase utilizers (log10) & Hydrogenase utilizers (log10)			
Hydrogenase utilizers (log10) & *L. salivarius* (log10)	-0.183165	-2.33455	0.020833
Hydrogenase utilizers (log10) & *C. jejuni* (log10)	0.498503	7.20534	0.000000
L. salivarius (log10) & Methanogenic archaea (log10)	0.049604	0.62230	0.534646
L. salivarius (log10) & Acetogens (log10)	-0.143439	-1.81606	0.071268
L. salivarius (log10) & Sulfate-reducing bacteria (log10)	0.263881	3.42791	0.000777
L. salivarius (log10) & Hydrogenase utilizers (log10)	-0.183165	-2.33455	0.020833
L. salivarius (log10) & *L. salivarius* (log10)			
L. salivarius (log10) & *C. jejuni* (log10)	0.034213	0.42894	0.668552
C. jejuni (log10) & Methanogenic archaea (log10)	0.472977	6.72632	0.000000
C. jejuni (log10) & Acetogens (log10)	0.014771	0.18511	0.853386
C. jejuni (log10) & Sulfate-reducing bacteria (log10)	0.431407	5.99177	0.000000
C. jejuni (log10) & Hydrogenase utilizers (log10)	0.498503	7.20534	0.000000
C. jejuni (log10) & *L. salivarius* (log10)	0.034213	0.42894	0.668552
C. jejuni (log10) & *C. jejuni* (log10)			
