# Supplementary material for: Microorganisms Involved in Hydrogen Sink in the Gastrointestinal Tract of Chickens
Source: Int J Mol Sci. 2023 Apr 3;24(7):6674. doi: 10.3390/ijms24076674 (PMC10095559; doi:10.3390/ijms24076674)
Supplement: Supplementary file 1 [file ijms-24-06674-s001.zip › table S2.pdf]

Pair of variables	Source=free-range group The Spearman rho's rank correlation results; $p < 0.05000$		
	R_s Spearman	t(N-2)	*p*
Methanogenic archaea (log10) & Methanogenic archaea (log10)			
Methanogenic archaea (log10) & Acetogens (log10)	0.383513	2.906866	0.005469
Methanogenic archaea (log10) & Sulfate-reducing bacteria (log10)	0.252893	1.829727	0.073378
Methanogenic archaea (log10) & Hydrogenase utilizers (log10)	0.099262	0.698284	0.488300
Methanogenic archaea (log10) & *L. salivarius* (log10)	-0.042357	-0.296764	0.767901
Methanogenic archaea (log10) & *C. jejuni* (log10)	0.039408	0.276069	0.783656
Acetogens (log10) & Methanogenic archaea (log10)	0.383513	2.906866	0.005469
Acetogens (log10) & Acetogens (log10)			
Acetogens (log10) & Sulfate-reducing bacteria (log10)	0.449429	3.521714	0.000938
Acetogens (log10) & Hydrogenase utilizers (log10)	0.185395	1.320662	0.192749
Acetogens (log10) & *L. salivarius* (log10)	0.003980	0.027858	0.977889
Acetogens (log10) & *C. jejuni* (log10)	0.201828	1.442481	0.155529
Sulfate-reducing bacteria (log10) & Methanogenic archaea (log10)	0.252893	1.829727	0.073378
Sulfate-reducing bacteria (log10) & Acetogens (log10)	0.449429	3.521714	0.000938
Sulfate-reducing bacteria (log10) & Sulfate-reducing bacteria (log10)			
Sulfate-reducing bacteria (log10) & Hydrogenase utilizers (log10)	0.414109	3.184663	0.002520
Sulfate-reducing bacteria (log10) & *L. salivarius* (log10)	0.068154	0.478187	0.634644
Sulfate-reducing bacteria (log10) & *C. jejuni* (log10)	0.390982	2.973573	0.004556
Hydrogenase utilizers (log10) & Methanogenic archaea (log10)	0.099262	0.698284	0.488300
Hydrogenase utilizers (log10) & Acetogens (log10)	0.185395	1.320662	0.192749
Hydrogenase utilizers (log10) & Sulfate-reducing bacteria (log10)	0.414109	3.184663	0.002520
Hydrogenase utilizers (log10) & Hydrogenase utilizers (log10)			
Hydrogenase utilizers (log10) & *L. salivarius* (log10)	0.570129	4.857742	0.000013
Hydrogenase utilizers (log10) & *C. jejuni* (log10)	0.760057	8.187047	0.000000
L. salivarius (log10) & Methanogenic archaea (log10)	-0.042357	-0.296764	0.767901
L. salivarius (log10) & Acetogens (log10)	0.003980	0.027858	0.977889
L. salivarius (log10) & Sulfate-reducing bacteria (log10)	0.068154	0.478187	0.634644
L. salivarius (log10) & Hydrogenase utilizers (log10)	0.570129	4.857742	0.000013
L. salivarius (log10) & *L. salivarius* (log10)			
L. salivarius (log10) & *C. jejuni* (log10)	0.305739	2.247810	0.029122
C. jejuni (log10) & Methanogenic archaea (log10)	0.039408	0.276069	0.783656
C. jejuni (log10) & Acetogens (log10)	0.201828	1.442481	0.155529
C. jejuni (log10) & Sulfate-reducing bacteria (log10)	0.390982	2.973573	0.004556
C. jejuni (log10) & Hydrogenase utilizers (log10)	0.760057	8.187047	0.000000
C. jejuni (log10) & *L. salivarius* (log10)	0.305739	2.247810	0.029122
C. jejuni (log10) & *C. jejuni* (log10)			
