# Supplementary material for: Microorganisms Involved in Hydrogen Sink in the Gastrointestinal Tract of Chickens
Source: Int J Mol Sci. 2023 Apr 3;24(7):6674. doi: 10.3390/ijms24076674 (PMC10095559; doi:10.3390/ijms24076674)
Supplement: Supplementary file 1 [file ijms-24-06674-s001.zip › table S3.pdf]

Pair of variables	Source=commercial group The Spearman rho's rank correlation results; $p < 0.05000$		
	R_s Spearman	t(N-2)	*p*
Methanogenic archaea (log10) & Methanogenic archaea (log10)			
Methanogenic archaea (log10) & Acetogens (log10)	0.641109	6.02398	0.000000
Methanogenic archaea (log10) & Sulfate-reducing bacteria (log10)	0.225229	1.66698	0.101531
Methanogenic archaea (log10) & Hydrogenase utilizers (log10)	0.248798	1.85235	0.069655
Methanogenic archaea (log10) & *L. salivarius* (log10)	0.225620	1.67003	0.100924
Methanogenic archaea (log10) & *C. jejuni* (log10)	0.227463	1.68441	0.098096
Acetogens (log10) & Methanogenic archaea (log10)	0.641109	6.02398	0.000000
Acetogens (log10) & Acetogens (log10)			
Acetogens (log10) & Sulfate-reducing bacteria (log10)	0.357692	2.76210	0.007920
Acetogens (log10) & Hydrogenase utilizers (log10)	0.164740	1.20441	0.233885
Acetogens (log10) & *L. salivarius* (log10)	0.275188	2.06410	0.044014
Acetogens (log10) & *C. jejuni* (log10)	0.200609	1.47663	0.145806
Sulfate-reducing bacteria (log10) & Methanogenic archaea (log10)	0.225229	1.66698	0.101531
Sulfate-reducing bacteria (log10) & Acetogens (log10)	0.357692	2.76210	0.007920
Sulfate-reducing bacteria (log10) & Sulfate-reducing bacteria (log10)			
Sulfate-reducing bacteria (log10) & Hydrogenase utilizers (log10)	0.138590	1.00912	0.317588
Sulfate-reducing bacteria (log10) & *L. salivarius* (log10)	0.210560	1.55319	0.126443
Sulfate-reducing bacteria (log10) & *C. jejuni* (log10)	0.144732	1.05478	0.296401
Hydrogenase utilizers (log10) & Methanogenic archaea (log10)	0.248798	1.85235	0.069655
Hydrogenase utilizers (log10) & Acetogens (log10)	0.164740	1.20441	0.233885
Hydrogenase utilizers (log10) & Sulfate-reducing bacteria (log10)	0.138590	1.00912	0.317588
Hydrogenase utilizers (log10) & Hydrogenase utilizers (log10)			
Hydrogenase utilizers (log10) & *L. salivarius* (log10)	-0.123593	-0.89813	0.373257
Hydrogenase utilizers (log10) & *C. jejuni* (log10)	0.259824	1.94026	0.057780
L. salivarius (log10) & Methanogenic archaea (log10)	0.225620	1.67003	0.100924
L. salivarius (log10) & Acetogens (log10)	0.275188	2.06410	0.044014
L. salivarius (log10) & Sulfate-reducing bacteria (log10)	0.210560	1.55319	0.126443
L. salivarius (log10) & Hydrogenase utilizers (log10)	-0.123593	-0.89813	0.373257
L. salivarius (log10) & *L. salivarius* (log10)			
L. salivarius (log10) & *C. jejuni* (log10)	-0.336411	-2.57604	0.012874
C. jejuni (log10) & Methanogenic archaea (log10)	0.227463	1.68441	0.098096
C. jejuni (log10) & Acetogens (log10)	0.200609	1.47663	0.145806
C. jejuni (log10) & Sulfate-reducing bacteria (log10)	0.144732	1.05478	0.296401
C. jejuni (log10) & Hydrogenase utilizers (log10)	0.259824	1.94026	0.057780
C. jejuni (log10) & *L. salivarius* (log10)	-0.336411	-2.57604	0.012874
C. jejuni (log10) & *C. jejuni* (log10)			
