# Supplementary material for: Microorganisms Involved in Hydrogen Sink in the Gastrointestinal Tract of Chickens
Source: Int J Mol Sci. 2023 Apr 3;24(7):6674. doi: 10.3390/ijms24076674 (PMC10095559; doi:10.3390/ijms24076674)
Supplement: Supplementary file 1 [file ijms-24-06674-s001.zip › table S4.pdf]

Pair of variables	Source=experimental group The Spearman rho's rank correlation results; *p* < 0.05000		
	R_s Spearman	t(N-2)	*p*
Methanogenic archaea (log10) & Methanogenic archaea (log10)			
Methanogenic archaea (log10) & Acetogens (log10)			
Methanogenic archaea (log10) & Sulfate-reducing bacteria (log10)			
Methanogenic archaea (log10) & Hydrogenase utilizers (log10)			
Methanogenic archaea (log10) & *L. salivarius* (log10)			
Methanogenic archaea (log10) & *C. jejuni* (log10)			
Acetogens (log10) & Methanogenic archaea (log10)			
Acetogens (log10) & Acetogens (log10)			
Acetogens (log10) & Sulfate-reducing bacteria (log10)			
Acetogens (log10) & Hydrogenase utilizers (log10)	-0.032453	-0.23414	0.815794
Acetogens (log10) & *L. salivarius* (log10)	0.188607	1.38492	0.171991
Acetogens (log10) & *C. jejuni* (log10)	0.189484	1.39160	0.169969
Sulfate-reducing bacteria (log10) & Methanogenic archaea (log10)			
Sulfate-reducing bacteria (log10) & Acetogens (log10)			
Sulfate-reducing bacteria (log10) & Sulfate-reducing bacteria (log10)			
Sulfate-reducing bacteria (log10) & Hydrogenase utilizers (log10)			
Sulfate-reducing bacteria (log10) & *L. salivarius* (log10)			
Sulfate-reducing bacteria (log10) & *C. jejuni* (log10)			
Hydrogenase utilizers (log10) & Methanogenic archaea (log10)			
Hydrogenase utilizers (log10) & Acetogens (log10)	-0.032453	-0.23414	0.815794
Hydrogenase utilizers (log10) & Sulfate-reducing bacteria (log10)			
Hydrogenase utilizers (log10) & Hydrogenase utilizers (log10)			
Hydrogenase utilizers (log10) & *L. salivarius* (log10)	-0.531545	-4.52525	0.000035
Hydrogenase utilizers (log10) & *C. jejuni* (log10)	-0.080068	-0.57924	0.564930
L. salivarius (log10) & Methanogenic archaea (log10)			
L. salivarius (log10) & Acetogens (log10)	0.188607	1.38492	0.171991
L. salivarius (log10) & Sulfate-reducing bacteria (log10)			
L. salivarius (log10) & Hydrogenase utilizers (log10)	-0.531545	-4.52525	0.000035
L. salivarius (log10) & *L. salivarius* (log10)			
L. salivarius (log10) & *C. jejuni* (log10)	0.074914	0.54173	0.590317
C. jejuni (log10) & Methanogenic archaea (log10)			
C. jejuni (log10) & Acetogens (log10)	0.189484	1.39160	0.169969
C. jejuni (log10) & Sulfate-reducing bacteria (log10)			
C. jejuni (log10) & Hydrogenase utilizers (log10)	-0.080068	-0.57924	0.564930
C. jejuni (log10) & *L. salivarius* (log10)	0.074914	0.54173	0.590317
C. jejuni (log10) & *C. jejuni* (log10)			
